# The Impact of Whole Sesame Seeds on the Expression of Key-Genes Involved in the Innate Immunity of Dairy Goats

**DOI:** 10.3390/ani11020468

**Published:** 2021-02-10

**Authors:** Christina Mitsiopoulou, Kyriaki Sotirakoglou, Dimitrios Skliros, Emmanouil Flemetakis, Eleni Tsiplakou

**Affiliations:** 1 Laboratory of Nutritional Physiology and Feeding, Department of Animal Science, School of Animal Biosciences, Agricultural University of Athens, Iera Odos 75, 118 55 Athens, Greece; chr_mitsiopoulou28@hotmail.com; 2 Laboratory of Mathematics and Statistics, Department of Natural Resources Management and Agricultural Engineering, Agricultural University of Athens, Iera Odos 75, 11855 Athens, Greece; sotirakoglou@aua.gr; 3 Laboratory of Molecular Biology, Department of Biotechnology, School of Food, Biotechnology and Development, Agricultural University of Athens, Iera Odos 75, 11855 Athens, Greece; dsklhros@gmail.com (D.S.); mflem@aua.gr (E.F.)

**Keywords:** whole sesame seeds, innate immunity, blood, neutrophils, goats

## Abstract

**Simple Summary:**

This study examined the impact of whole sesame seeds (WSS), rich in both linoleic acid and lignans, on the innate immunity of goats. WSS were incorporated in the concentrates of the control group at 5 and 10% respectively, by partial substitution of both soybean meal and corn grain. The highest supplementation level of WSS resulted in a significant down-regulation in the expression levels of several pro-inflammatory genes in the neutrophils of goats. In conclusion, the dietary supplementation of goats with WSS might be a good nutritional strategy to improve their innate immunity.

**Abstract:**

Whole sesame seeds (WSS) are rich in both linoleic acid (LA) and lignans. However, their impact on the innate immunity of goats is not well studied. Twenty-four goats were divided into three homogeneous sub-groups; comprise one control (CON) and two treated (WWS5 and WWS10). In the treated groups, WSS were incorporated in the concentrates of the CON at 5 (WSS5) and 10% (WSS10) respectively, by partial substitution of both soybean meal and corn grain. The expression levels of *MAPK1*, *IL6*, *TRIF*, *IFNG*, *TRAF3*, and *JUND* genes in the neutrophils of WSS10 fed goats were reduced significantly compared with the CON. The same was found for the expression levels of *IFNG* and *TRAF3* genes in the neutrophils of WSS5 fed goats. Both treated groups primarily affected the MYD88-independent pathway. The dietary supplementation of goats with WSS might be a good nutritional strategy to improve their innate immunity.

## 1. Introduction

N-3 polyunsaturated fatty acids (PUFA) in humans have anti-inflammatory role [1] since resolve inflammation [2] and eliminate pain in inflammatory circumstances [3]. On the other hand, increased consumption of linoleic acid (LA), the main fatty acid (FA) of the n-6 PUFA group, might be related with inflammatory diseases due to its metabolization in LA-derived pro-inflammatory lipoxins and arachidonic acid, which further leads to pro-inflammatory eicosanoids and prostaglandins production [4]. An enhancement in the concentrations of pro-inflammatory leukotriene and prostaglandins [5] was found in rats fed with high LA diets. Significantly higher tumor necrosis factor a (TNFA), and interleukin-7 concentrations in the liver of pregnant rat, consumed a high compared with low LA diet, was observed, without the cytokines content in their blood to be affected [6]. Similarly, excessive dietary LA consumption increased significantly the TNFA content in plasma and nuclear factor-kappa B (*NF-KB*) expression in rats’ aortas [7]. On the other hand, recent reviews and meta-analysis studies provide evidence that LA intake decreases [8] or has no effect on cardiovascular diseases [9,10] disputing its role in chronic diseases involving inflammatory process.

So far, to the best of our knowledge, no information exists on the impact of LA in the innate immunity of productive animals, and particularly in goats. Thus, whole sesame seeds (WSS), due to their high LA (44%) content [11], can be used in goats’ diets to test this hypothesis. Moreover, WSS contain lignans such as sesamin and sesamolin, which might have several beneficial effects in immunity [12]. The anti-inflammatory properties of sesame in rats’ models through in vitro and in vivo trials have been reviewed recently [13]. Sesamin down-regulates the expression of Toll-like receptor 4 (*TLR4*) gene in lipopolysaccharide (LPS) stimulated BV-2 microglial cell line of rats, in a dose dependent matter in vitro [14]. Accordingly, 50 μM of sesamin suppressed the activation of p38 mitogen-activated protein kinase (MAPK) signaling pathway after its stimulation with LPS [15]. A significant decline in the expression of interleukin 1 Beta (*IL1B*), interleukin-2 (*IL2*) and *TNFA* genes in mouse senescence-accelerated brain cells was found, when fed with sesaminol [16]. So far, the impact of dietary inclusion of WSS in the immune system of produced animals has not been studied.

The immune system is broken down into innate and adaptive [17]. Neutrophils comprise one of the main cellular component of the innate immune system and the first line of defense against pathogens [18]. The innate immune system employs special receptors known as pattern-recognition receptors (PRRs) such as NOD-like receptors (NLRs) that recognize pathogen- or damage-associated molecular patterns (PAMPs and DAMPs, respectively) [19]. Among the PRRs, Toll-like receptors (TLRs) enact the induction of immune response [20]. All TLR signaling pathways end up in activation of the transcription factor nuclear factor-kappa B (*NF-KB*) and interferons (IRFs), which regulate the outcome of innate immune responses [19]. In addition, a core element of the NF-KB cascade is the IκB kinase (*IKK*) complex or conserved helix-loop-helix ubiquitous kinase (CHUK) which is encoded by the *CHUK* gene [21]. TLR activation stimulates the release of various inflammatory cytokines (*TNF*A, interferon *IFNG*, interleukins (*IL1B*, *IL2* and *IL6*) and immune modulators such as IL8, C-C motif chemokine ligand 5 (*CCL5)* and chemokine (C-X-C motif) ligand 16 (CXCL16) [22,23,24]. After that, *IL6* induces downstream signaling of the signal transducer and activator of transcription 3 (*STAT3*) [25]. Upon PAMPs and DAMPs recognition, TLRs recruit Toll-interleukin-1 receptor TIR domains, which transmit downstream signals via adaptor molecules such as myeloid differentiation primary response gene 88 (*MΥD88*) and the TIR (Toll/Interleukin-1 Receptor) domain-containing adaptor protein inducing interferon beta (TRIF) [26,27]. The MYD88-dependent pathway activates the *IRF5* gene expression [28] and the pathway involving mitogen-activated protein kinases (MAPKs). TNF Receptor-associated Factor 3 (*TRAF3*) is incorporated into both MYD88 and TRIF complex, activating MYD88-dependent signaling and suppressing TRIF-dependent pathway [29]. TRAF3 mediates activation of *IRF3* [30]. The extracellular signal regulated kinase (ERK)–mitogen-activated protein kinase pathway determines the regulation of *JUND* gene expression [31]. Finally, Heme Oxygenase-1 (*HO1*) gene has the ability to modulate immune responses [32].

Taking into account all the above, the objective of this study was to investigate the effects of dietary inclusion of WSS at two different levels (5 and 10%) on the expression of selected key-genes (*NLRC3*, *TLR4*, *MYD88*, *NF-KB*, *MAPK1*, *IL1A*, *IL1B*, *TNFA*, *TNFB*, *IL2*, *IL6*, *IL10*, *STAT3*, *TRIF*, *IRF3*, *IFNG*, *TRAF3*, *IRF5*, *CCL5*, *IL8*, *CXCL16*, *HO1*, *JUND* and *CHUK*) involved in the innate immunity of dairy goats.

## 2. Materials and Methods

### 2.1. Animals and Diets

Animal handling procedures were performed in accordance with protocols approved by the Agricultural University of Athens Ethical Committee of the Faculty of Animal Sciences. Twenty-four goats were divided into three homogenous subgroups (*n* = 8) according to their fat-corrected milk yield (1.00 ± 0.22 kg/day) and body weight (44.9 ± 5.4 kg). The goats were fed on a group basis with a basal diet consisted of alfalfa hay, wheat straw and concentrates (Forage/Concentrate ratio = 50/50), for a seven-day adaptation period. The forages were provided separately from the concentrates while they were both offered to the animals twice a day (in two equal parts at 08:00 and 18:30 h) after milking. After the adaptation period the control goats continued to consume the basal diet, in the concentrates of which hulled sesame seeds were not included (CON). On the other hand, in the concentrates of the two other groups whole sesame seeds at 5 (WSS5) and 10% (WSS10) respectively, were incorporated by partial substitution of both soybean meal and corn grain (Table 1), in order the dietary treatments to be iso-energetic and iso-protein, and to meet the animals’ average maintenance and lactation requirements [33]. The quantities of food offered to the animals were adjusted every two weeks, according to their average requirements, based on their body weight and milk fat-corrected yield. Diet selectivity did not occur, and no refusals of forage and/or concentrates were observed. The mineral and vitamin premix of both concentrates contained the following (per kg as mixed): 150 g Ca, 100 g P, 100 g Na, 100 mg Co, 300 mg I, 5000 mg Fe, 10,000 mg Mn, 20,000 mg Zn, 100,000 mg Se, 5,000,000 IU retinol, 500,000 IU cholecalciferol and 15,000 mg α-tocopherol. The experimental period, lasted 100 days and all the animals had free access to fresh water.

### 2.2. Feed Sample Analyses

Samples of the alfalfa hay, wheat straw and concentrate were analyzed for organic matter (OM; Official Method 7.009), dry matter (DM; Official Method 7.007) and crude protein (CP; Official Method 7.016) according to the AOAC (1984) and for neutral detergent fiber (NDF) and acid detergent fiber (ADF)-expressed exclusive of residual ash-according to the methods of Van Soest et al. [34].

### 2.3. Blood Samples

#### 2.3.1. Blood Sample Collection for Neutrophil Isolation

Blood samples were taken at the 30th, 60th and 90th day from the beginning of the experiment for neutrophil isolation from the jugular vein into 17 Units/mL heparine-containing tubes (BD Vacutainer, Plymouth, UK). 

#### 2.3.2. Cell Isolation

Cell isolation was performed according to Tsiplakou et al. [35]. More specifically, isolation of neutrophils is carried out using density gradient centrifugation Histopaque 1077 (Sigma-Aldrich, St. Louis, MO, USA). Analytically, whole blood mixed with an equal volume of Hanks’ balanced salt solution and three parts diluted blood layered on two parts Histopaque. Samples were centrifuged for 40 min at 500× *g*, 4 °C with the minimum acceleration and deceleration. After centrifugation, the upper phases were rejected. Neutrophils cells, which remained in the red cell layer, were lysed with the addition of endotoxin-free ultrapure water, and were vigorously shaken. NaCl was then added to resuspend cells in an isotonic solution (0.9% NaCl). These cells were washed several times and centrifuged for 5 min at 1000 g and 4 °C until a white and consistent cell pellet was clearly visible at the bottom of the tube. In the sequel, the final cell suspensions were cultured in 1 mL of growth medium RPMI (Sigma-Aldrich, St. Louis, MO, USA) which is incubated at 37 °C and then centrifuged at 1000 g for 5 min at 4 °C. Finally, the resulting cell pellets were again washed at least twice in 0.5 mL of phosphate-buffered saline (PBS) and centrifuged at 700 rpm for 1 min at 4 °C.

#### 2.3.3. RNA Extraction

The isolated cells were homogenized with TRIzol™ (Invitrogen, Carlsbad, CA, USA) and after centrifugation with 24:23:1 phenol: chloroform: isoamyl alcohol solution, three distinct layers were obtained. The upper clear aqueous phase containing the RNA was transferred carefully into a new tube, without disturbing the interphase. RNA pellet was precipitated with 70% ethanol and then was dissolved in milli-Q water. The quantity and quality of the extracted RNA were evaluated by ND-1000 spectrophotometer (NanoDrop, Wilmington, DE, USA); the quantity was measured in ng/μL, and its purity was determined based on the A260/A280 and A260/A23 ratios. In addition, RNA integrity was assessed by electrophoresis on an agarose gel. As defined, the isolated RNA was treated with Turbo™ DNase I (2U/μL, commercially available kit: Invitrogen, Carlsbad, CA, USA), accordingly to the manufacturer’s instructions. Absence of genomic DNA contamination was confirmed by PCR, using glycer aldehyde3-phosphatedehydro genase (GAPDH; housekeeping gene) Then, RNA samples were further purified by using phenol: chloroform and ethanol precipitation. The quantity and quality of the pure RNA samples were again confirmed by spectrophotometry (NanoDropND-1000) and by agarose gel (0.7%) electrophoresis.

#### 2.3.4. cDNA Synthesis

Approximately 500 ng of RNA was used per cDNA synthesis by using the Prime Script First Strand cDNA Synthesis Kit (Takara, Shiga, Japan) according to the manufacturer’s protocol using a mix of random hexamers and oligo-dT primers. 

#### 2.3.5. Primers

To derive primers sequences the ARS1 goat annotation was used. A pair of primers specific for each target gene (Table 2) were used by previous studies [35,36] designed to be specific for *Capra hircus* by using Primer Express Software (version 3.0) and verified using the Geneious Software (Biomatters, Auckland, New Zealand) and were tested against genomic DNAs to confirm that a single amplicon of 70 bp would result from quantitative real-time PCR (qPCR). In addition, dissociation curves were generated, and the amplification products were subjected to agarose gel electrophoresis to confirm the production of a single amplicon per reaction.

#### 2.3.6. Real-Time Quantitative PCR

The expression levels of genes were estimated by a Step One Plus ™ Real-Time PCR System (Applied Biosystems, Foster City, CA, USA) using SYBR Select Master Mix (Applied Biosystems, Austin, TX, USA), gene-specific primers at a final concentration of 0.2 μM each (forward and reverse) and 1 μL of each cDNA as template. Thermal cycling was started with denaturation at 95 °C for 15 min, followed by 40 cycles at 95 °C for 15 s and 62 °C for 10 s. GAPDH and YWHAZ were used as housekeeping genes to normalize the cDNA template concentrations. The choice of housekeeping genes was based on a study by Vorachek et al. [38].

#### 2.3.7. Normalization

The expression levels of the genes were calculated as (1 + E)^−ΔCt^, where ΔCt is the difference between the geometric mean of the two housekeeping genes’ Cts and the Ct of the target gene, and the primer efficiency is the mean of each amplicon’s efficiency per primer, which was calculated by employing the linear regression method on the log (fluorescence) per cycle number (ΔRn) using the LinReg PCR software [37].

#### 2.3.8. Statistical Analysis

Experimental data are presented as least squares means ± standard errors and were analyzed using a general linear model (GLM) for repeated measures, considering the sampling time (T) as the repeated measure, with fixed effects of dietary treatments (D) (CON, WSS5, WSS10), sampling time (T) (30th, 60th, 90th experimental day) and the interactions among them (D × T) according to the model:

Yijk = μ + Di + Tj + (D × T) ij + Ak + eijk(1)

where Υijk is the dependent variable, μ the overall mean, Di the effect of dietary treatment (I = 1, 2, 3), Tj the effect of sampling time (j = 1, 2, 3), (D × T) ij the interaction between dietary treatments and sampling time, Ak the animal’s random effect and eijk the residual error. Post hoc analyses were performed when appropriate using Duncan’s multiple range test. Kolmogorov-Smirnov test revealed that all variables followed a normal distribution. Pearson’s correlation coefficients were used to determine the relationships between gene expression in neutrophils using heat map chart. For all tests, the significance was set at 0.05. Graphs were drawn using SPSS software (version 20.0, IBM, Armonk, NY, USA), and the error bars represent the standard error of the mean (SEM). Statistical analysis was performed using the statistical packages SPSS software (version 20.0, IBM, Armonk, NY, USA).

## 3. Results

**Figure 1:**
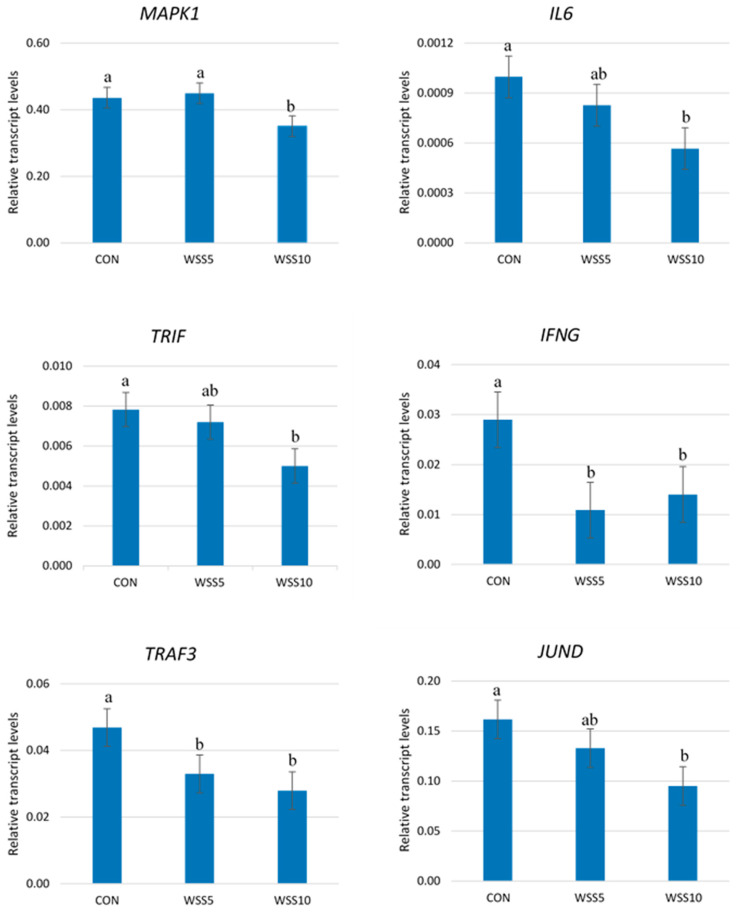
The Transcript abundance of several genes in the neutrophils of goats. Bars represent means ± SEM of each (*n* = 8) of the three dietary treatments; CON: control, basal diet; WSS5: basal diet + 5% whole sesame seed; WSS10: basal diet + 10% whole sesame seed in goats. For each gene, bars with different superscripts (a, b) between the three dietary treatments (CON, WSS5, WSS10) differ significantly (*p* ≤ 0.05), according to the analysis of variance (ANOVA) using a general linear model (GLM) for repeated measures. Post hoc analysis was performed using Duncan’s multiple range test.

A significant reduction in the expression levels of *MAPK1*, *IL6, TRIF, IFNG*, *TRAF3* and *JUND* genes in the neutrophils of WSS10 fed goats compared with the CON was found (Figure 1).

The same trend was found for the IFNG and TRAF3 genes in the neutrophils of SS5 fed goats (Figure 1). No differences were found in the expression levels of the above genes between the treated groups (Supplementary Figure S1).

A significant reduction in the expression levels of *NF-KB*, *MYD88*, *MAPK1*, *TNFA,* and *STAT3* genes in the neutrophils of goats throughout the experimental period was observed (Supplementary Table S1). The opposite happened in the relative abundance transcripts of *IRF5* and *HO1* genes (Supplementary Table S1). The highest expression levels of *NLRC3*, *TNFB* and *TRIF* genes were indicated in the 60th experimental day while in this day the *TLR4* gene showed the lowest expression levels (Supplementary Table S1). A significant decline in the expression levels of *IL1A*, *ΙL-2* and *CCL5* genes was found in the 90th compared with the 30th and 60th experimental period, while the opposite trend was observed for the *IL10* and *CXXL-16* genes (Supplementary Table S1).

**Figure 2:**
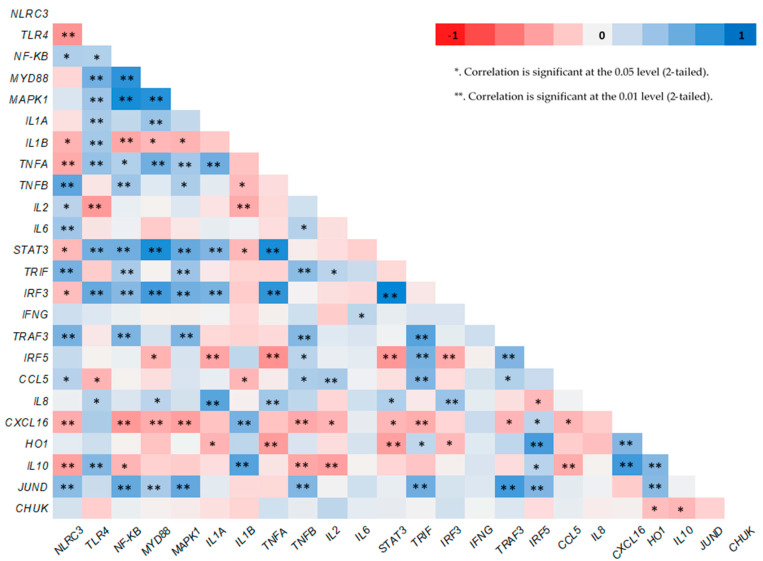
Pearson’s heat map correlations between the expression level of several genes in neutrophils of goats.

Significantly positive correlations between the expression levels of; *TLR4* with *NF-KB*, *MYD88*, and *IL1B*, *MYD88* with *NF-KB* and *IRF3*, *STAT3* with *MYD88* and *NF-KB*, *JUND* with *HO1*, *NF-KB* with *IRF3* genes respectively, as well as between *MAPK1* with *MYD88* and *TLR4* and *NLRC3* with *IL6* and *TNFB* genes respectively, were found (Figure 2). 

## 4. Discussion

The impact of sesame seeds in the innate immunity of ruminants has rarely been investigated to the best of our knowledge. Neutrophils are involved in initiation of the inflammatory response [39], through the expression of several families of PRRs such as NLRs and TLRs [40,41] which can identify either microbial pathogens or components of host’s cells that are released during cell damage or death. Significantly higher expression levels of *TLR4* gene have been found in blood neutrophils of ketotic cows [42]. Moreover, Roldan-Montes et al. [43] found in milk, a significant association between the identified polymorphisms of the *TLR4* gene and the somatic cell score of water buffaloes. A significant down-regulation in the expression of *TLR4* gene in LPS-stimulated BV-2 microglial cell line of rats was observed in vitro [14]. Thus, the results of this study referring to *NLRC3* and *TLR4* genes (Supplementary Figure S1) might show not only absence of any inflammation (clinical or subclinical) but also the protective role of sesame seeds in goats’ cells survival. 

*TLR4* gene regulates the NF-KB pathway through the *MYD88* gene [44] which affects the MAPKs cascade [45]. Indeed, significantly positive correlations between the expression levels of *TLR4* with *NF-KB*, *TLR4* with *MYD88* and *MYD88* with *NF-KB* genes, were found (Figure 2). It was observed that sesamin, one of the main antioxidant compounds of sesame seeds, reduces the activation of NF-KB (measured by ELISA) and P38 MAPK kinase (measured by Western blot) in mice microglia cells treated with LPS [15]. The same has been shown for the expression of *TLR4* gene (measured by flow cytometry) in hepatic tissue of mice [46]. Sesamin inclusion in RPMI-8226 cells [47] and sesame oil aqueous extract in RAW 264.7 macrophages of mice treated with LPS [48] down regulate the expression of *NF-KB* gene in vitro. Additionally, a significant up-regulation in expression levels of *TLR4* and *MYD88* genes in goats’ mammary epithelial cells [49], and in human endometrial cells [50] when stimulated in vitro by LPS have been found. Moreover, significantly higher expression levels of *MYD88* gene have been observed in bovine mastitis tissue [51]. Thus, the results of this study, concerning the *NF-KB*, *MYD88* and *MAPK1* genes, not only show no pathogens, stress or endogenous inflammatory factors in goats’ organisms but also indicate a positive effect in their innate immunity when the animals were fed with the higher supplementation level (10%) of WSS.

NLRs and TLRs stimulate the MAPKs cascade thought the MYD88 pathway [52] and trigger the cytokines production [53,54]. The significantly positive correlations between the expression levels of *MAPK1* with *MYD88* and *TLR4* genes respectively, confirm this close relationship (Figure 2). Moreover, the positive correlations between the expression levels of *TLR4* and *IL1B* genes, as well as between the expression levels of *NLRC3* with *IL6* and *TNFB* genes respectively (Figure 2) show that both NLRs and TLRs regulate the cytokines expression. IL2 has anti-inflammatory properties [55] while IL6 is elevated in most cases of inflammation and have been recognized as target for therapeutic intervention [56]. Indeed, it has been found recently that elevated IL6 levels in blood plasma resulted a STAT3 hyperactivation in tumor cells [25]. However, sesamin has the ability to suppress the STAT3 signaling pathway (IL6/JAK/STAT3) in human hepatocellular carcinoma cell line HepG2 [57]. The anti-cancer effects of sesamin have been attributed to its ability to reduce significantly the expression of *NF-KΒ*, *IL6* and transcriptional target of *STAT3* [58]. In accordance with our findings, sesamin inhibits the expression levels of *IL6* gene, in a dose depend matter in vitro [15]. The anti-inflammatory activities of sesamin have been shown also, in influenza H1N1-induced peripheral blood mononuclear cells of humans by either the reduction in the expression levels of both *IL1B* and *TNFA* genes or the increase in the expression of *IL2* gene [59]. A significant reduction in the expression levels of IL2 gene was observed in cows infected with malignant catarrhal fever [60,61]. Furthermore, a significant down-regulation in the expression levels of pro-inflammatory (*IL1A, IL1B, TNFA*) genes including *IL6* was found in the liver of mice fed with a sesame oil rich diet [62]. Sesamin, reversed the inflammation which caused by the consumption of a high fat diet in rats by reducing the expression of *IL6* and *TNFA* genes [63]. Thus, the results of this study, as the *IL2*, *IL6*, *STAT3* and cytokines genes expression is concerned could high light as well the idea of an improvement of goats’ innate immunity especially, when they were fed with the higher inclusion level of sesame seeds.

Although MYD88 is a common adaptor for all the TLRs except TLR3, TRIF, is an adaptor for TLR3 and TLR4 which promotes an alternative pathway that leads to the activation of IRF3 for induction of type IFN [19,64]. Our results referring to the *TRIF*, *IRF3* and *IFNG* genes, show that the highest dietary inclusion level (10%) of WSS affected also the MYD88 independent pathway. This is further supported by the changes in the expression levels of *TRAF* gene since both MYD88 and TRIF pathways are controlled by TRAF regulators such as TRAF3 [65,66]. Similar to our findings, a significant down-regulation in the expression levels of *IFNG* gene in cultured mononuclear cells of experimental autoimmune encephalomyelitis mice, fed with sesame oil, has been found [67]. Additionally, sesame oil reduces significantly the concentrations of IFNG in multiple sclerosis patients [68]. On the other hand, the expression level of *IRF3* gene increased significantly in goats’ mammary epithelial cells after 3 h incubation in vitro with both toxins from LPS and gram-positive lipoteichoic acid bacterial [49]. The same was observed in bovine mammary epithelial cells when stimulated either with *Escherichia coli* or *Staphylococcus aureus* [69]. Furthermore, the pro-inflammatory role of IRF3 has been indicated also in mice macrophages, through the activation of TLR4-TRIF metabolic pathway which regulates the production of pro-inflammatory cytokines [70].

Chemokines such as CCL5, IL8 and CXCL16 can be produced by many cells including neutrophils [71] after proper stimulation [72]. So far, significant higher expression levels of *CCL5* gene have been observed in infected blood macrophages with *Mycobacterium* in vitro [73]. The same trend has been indicated in goats’ mammary epithelial cells after incubation with gram-negative and/or gram-positive bacteria cell wall components in vitro [49]. The expression of *IL8* gene enhanced significantly in blood neutrophils of calving cows with clinical mastitis [71]. A positive correlation between *IL8* gene expression and the incidence of severe mastitis has been also shown [74]. However, chemokines such as IL8 can be also released from the cells as response to the reactive oxygen species (ROS) [75]. Additionally, CXCL16 chemokine can have a scavenger role for the uptake of oxidase molecules such as the low-density lipoproteins [76]. It has been shown that various antioxidant compounds can protect low-density lipoprotein (LDL) from oxidation in vitro [77]. Indeed, a delay in the oxidation of lipoproteins in the blood plasma of mice fed with sesame oil has been found due to its sesamin and sesamone content which was accompanied by a significant reduction in the CXCL16 blood plasma content [62]. Thus, the results of this study, concerning the expressions of chemokines (*CCL5*, *IL8* and *CXCL16*), further support the use of sesame seeds as a nutritional tool for the improvement of goats both innate immunity and antioxidant status.

*HO1* is a highly inducible gene well known for its anti-inflammatory, immunomodulatory and antioxidants functions [78]. Similar with our findings, sesamin did not modify the expression levels of *HO1* gene in rats in vitro [79]. On the contrary, a significant up-regulation in the expression levels of *HO1* gene has been found in the liver of bovine and mice, infected by *Fachiola hepatica* [80]. The same was observed for both *HO1* and *IL10* genes in LPS-stimulated macrophages of mice [81]. Although the metabolic pathway which regulates *HO1* gene expression in not clear, activation of STAT3 by IL10 cytokine has repeatedly been suggested. The positive relationship between the expression levels of *HO1* and *IL10* genes (*p* < 0.01) supports this suggestion while the negative relation between the expression levels of *STAT3* and *IL10* genes (*p* < 0.01), which was found in this study (Figure 2), needs further investigation in order to clarify the role of *STAT3* gene in this metabolic pathway. Thus, referring to the results on the expression levels of *HO1*, *IL10* and *STAT3* genes of this study, an enhancement of the innate immune responses with the higher supplementation level of WSS could be claimed.

*JUND* gene might have also an involvement in the *HO1* gene expression. It has been found that JUND protein repressed *HO1* gene expression in human renal epithelial cells [82]. The relationship between the expression levels of *HO1* and *JUND* genes supports this link (Figure 2). Moreover, the expression levels of *JUND* gene followed the same trend with the expression level of *MAPK1* gene (Supplementary Table S1). *JUND* gene has a fundamental role in the defense against oxidative stress [83]. Thus, its sharpest down-regulation with the highest supplementation level of sesame seeds might show that the SS10 goats had a sufficient pool of antioxidants compounds in their organism such as sesamin, sesaminol, etc. which enhance their innate immunity.

So far, in the innate immunity, little attention has been given in inflammatory mediators such as the IkappaB kinase (IKK). Τhe role of IKK-α subunit (*CHUK*) in inflammation is not well known. However, CHUK gene is required for the activation of the “alternative” NF-KB pathway which is activated by the TNF family cytokines [84]. Moreover, CHUK has anti-inflammatory role through the regulation of SUMO (small ubiquitin-related modifier) ligase activity of protein inhibitor of activated STAT1 (PIAS) [85]. In accordance with our findings sesamin had no effect on the expression of *CHUK* gene in various human cells lines in vitro [47]. More research is needed in order to clarify the role of *CHUK* gene in the innate immunity.

## 5. Conclusions

Overall, our study provides new evidence regarding the impact of dietary supplementation with WSS in the innate immunity of dairy goats. The highest inclusion level (WSS10) seems the best modulator of goats’ innate immunity, as demonstrated by the sharpest decline in the expression levels of genes (*MAPK1*, *IL6*, *TRIF*, *IFNG*, *TRAF3*, and *JUND*) involved with inflammatory metabolic pathways. The topmost intake of WSS also regulates both MYD88 dependent (*MAPK1*) and independent (*TRIF*, *TRAF3*, *IFNG*) pathway, while this of WSS5 the independent one only. The above findings are very important in animal husbandry since inflammation should be limited as much as possible, and animals’ innate immunity should be activated only when is needed in order to be stronger and more effective. Finally, lignans can eliminate the pro-inflammatory compound, which is produced by LA’s metabolism, making WSS one of the best way to administer LA in goats’ diet.

## Figures and Tables

**Table 1:** Nutrients and fatty acids intake from forages and concentrates, and the total antioxidant capacity and phenolic content of concentrates only.

Daily Nutrients Intake (g/goat)	Diets (Forages and Concentrates)		
	CON ^1^	WSS5 ^2^	WSS10 ^3^
Dry matter	2028.4	2027.6	2035.6
Ash	143.4	147.6	153.3
Ether extract	44.7	72.7	100.9
Crude protein	323.6	323.2	334.6
NDF ^4^	766.1	795.1	782.7
ADF ^5^	504.1	518.9	513.5
Daily Fatty Acids Intake (g/goat)			
C_14:0_	0.34	0.34	0.33
C_15:0_	0.13	0.14	0.14
C_16:0_	7.96	10.27	14.06
C_16:1(n-7)_	0.25	0.29	0.34
C_17:0_	0.26	0.27	0.21
C_18:0_	1.70	2.52	4.48
C_18:1(n-9)_	8.70	20.18	31.19
C_18:2(n-6)c_	20.28	33.42	44.58
C_20:0_	0.15	0.22	0.34
C_18:3(n-3)_	3.77	3.87	3.94
C_20:2_	0.10	0.09	0.10
C_22:0_	0.31	0.34	0.39
C_23:0_	0.10	0.10	0.10
C_22:2_	0.01	0.01	0.01
C_20:5(n-3)_	0.04	0.04	0.04
C_24:0_	0.46	0.48	0.50
C_24:1(n-9)_	0.14	0.14	0.14
	Concentrates		
Total Antioxidant Capacity	CON ^1^	WSS5 ^2^	WSS10 ^3^
FRAP ^6^ (μM ascorbic acid/g DM)	9.19	13.88	17.69
DPPH(% Inhibition)	41.89	51.24	49.91
Total phenolic content			
Folin-Ciocalteu (mg GAE/g DM)	63.12	94.30	126.03

^1^ CON: Control. ^2^ WSS5: Whole sesame seeds at 5%. ^3^ WSS10: Whole sesame seeds at 10%.^4^ NDF: Neutral detergent fiber. ^5^ ADF: Acid detergent fiber ^6^ FRAP: Ferric reducing ability of plasma.

**Table 2:** Primers used for real-time qPCR and the mean PCR efficiency for each gene as calculated by LinRegPCR software [37].

Gene	Forward Primer 5′-3′	Reverse Primer 5′-3′	Ensemble
*NLRC3*	CAACCTACTCCACGACCAGG	TGGATGAAGTTCCACTGCA	ENSG00000167984
*TLR4*	ATGAACCACTCCACTCGCTC	TCTTGCTCCTTAGAGGCCGT	ENSG00000136869
*NF-KB*	AAGCTGTGGTGGAGGACTTG	ACAGAGTTACCCAAGCGGTC	ENSG00000109320
*MYD88*	ACAGACAAACTATCGGCTGA	CACCTCTTCTCAATGAGTTCA	ENSG00000172936
*MAPK1*	GCAACGACCACATCTGCTAC	AGGTTGGAAGGCTTGAGGTC	ENSG00000100030
*IL1A*	TCAAGCCCAGATCAGCACAT	TGATTGAGGGCGTCGTTCAG	ENSG00000115008
*IL1B*	TGGATAGCCCATGTGTGCTG	CAGAACACCACTTCTCGGCT	ENSG00000125538
*TNFA*	GGGAGACACAAACTAAGGGCT	AACCTGCAGTTCAGCTCCG	ENSG00000232810
*TNFB*	ACTCCCGAAGCCCTTCACCCG	GGCGGAGGAAGGCGCGGTCCG	ENSG00000226979
*IL2*	AAATCCCGAGAACCTCAAGCT	TGTAGCGTTAACCTTGGGCA	ENSG00000109471
*IL6*	CAGCAAGGAGACACTGGCAGA	TCCATCTTTTTCCTCCATTTTTGG	ENSG00000136244
*STAT3*	CGCAATTAGGCAGAGCAACTG	CCCTGTATCAGAGACCATCCCA	ENSG00000168610
*TRIF*	GCACGTCTAGCCTGCTTAC	TTGCGGGCCCGCAGCATCT	ENSG00000127666
*IRF3*	CCAGAGGCTGGGGCACTGCC	CCTTCGGGACCTCGCCGTCA	ENSG00000126456
*IFNG*	AAATTCCGGTGGATGATCTG	ACCATTACATTGATGCTCTCC	ENSG00000111537
*TRAF3*	TAACTGCTGCATTCGCTCCA	GGAACACAAAGCTGGGGTTG	ENSG00000131323
*IRF5*	ACATCCCCAGTGAGAAGCAG	ATGGCATACAGATCCTGGCC	ENSG00000128604
*CCL5*	CAAGTGCTCCATGGCAGCAG	GTTGGCGCACACCTGACG	ENSG00000271503
*IL8*	CCTGCTCTCTGCAGCTCTGTG	TGCATTGGCATCGAAGTTCTG	ENSG00000169429
*CXCL16*	GTGCCTGTGTTGTCCCTCTT	GCTTGCACACCACGTAGAGT	ENSG00000161921
*HO1*	GAGCTGACCCGAGAAGGTTT	AGACGGGGTTCTCCTTGTTG	ENSG00000100292
*IL10*	CTGGGGGAGAAGCTGAAGAC	CTCTCTTCACCTGCTCCACC	ENSG00000136634
*JUND*	ACGCAGTTCCTCTTTCCCAA	CCAGCTGGTTTTGCTTGTGT	ENSG00000130522
*CHUK*	TGCAGGGAAAGAGGCAGAAA	GACCGAGCAGAACTCTGTGT	ENSG00000213341
*GAPDH*	AAAGCCATCACCATCTTCCA	ACCACGTACTCAGCACCTCAT	ENSG00000111640
*YWHAZ*	TGTTCTATTGTGCCTAGTACACTGT	CATCAAGACTCACTGCCTCCC	ENSG00000164924

## Data Availability

Not applicable.

## Supplementary Materials

The following are available online at https://www.mdpi.com/2076-2615/11/2/468/s1, Figure S1: The Transcript abundance of several genes in the neutrophils of goats. Bars represent means ± SEM of each (*n* = 8) of the three dietary treatments; CON: control, basal diet; WSS5: basal diet + 5% whole sesame seed; WSS10: basal diet + 10% whole sesame seed in goats. The analysis of variance (ANOVA) using a general linear model (GLM) for repeated measures revealed that for these genes there was not significant difference between the three dietary treatments (*p* > 0.05), Table S1: Transcript abundance of several genes in the neutrophils of goats: NOD-like receptor (*NLRC3*), Toll-like receptors 4 (*TLR4*), Nuclear factor kappa B (*NF-KB*), Myeloid-Differentiation-primary response gene 88 (MYD88), Mitogen-Activated Protein Kinase-1 (*MAPK1*), Interleukin 1 Alpha (*IL1A*), Interleukin 1 Beta (*IL1B*), Tumor necrosis factor Alpha (*TNFA*), Τumor necrosis factor Beta (*TNFB*), Interleukin 2 (*IL2*), Interleukin 6 (*IL6*), Signal Transducer and Activator of Transcription 3 (*STAT3*), TIR (Toll/Interleukin-1 Receptor) domain-containing adaptor protein inducing interferon beta (*TRIF*), Interferon Regulatory Factor 3 (*IRF3*), Interferon gamma (*IFNG*), TNF Receptor-associated Factor 3 (*TRAF3*), Interferon Regulatory Factor 5 (*IRF5*), C-C motif chemokine ligand 5 (*CCL5*), Interleukin 8 (*IL8*), Chemokine (C-X-C motif) ligand 16 (*CXCL16*), Heme Oxygenase-1 (*HO1*), Interleukin 10 (*IL10*), Transcription factor JunD (*JUND*) and Conserved Helix-Loop-Helix-Ubiquitous Kinase (*CHUK*) or IKKA relative to the geometrical mean of the references genes (Glyceraldehyde 3-Phosphate Dehydrogenase (*GAPDH*) and Tyrosine 3-monoxygenase/tryptophan 5-monooxygenase activation protein, zeta polypeptide (*YWHAZ*)).
