# Supplementary material for: The Impact of Whole Sesame Seeds on the Expression of Key-Genes Involved in the Innate Immunity of Dairy Goats
Source: Animals (Basel). 2021 Feb 10;11(2):468. doi: 10.3390/ani11020468 (PMC7916339; doi:10.3390/ani11020468)
Supplement: Supplementary file 1 [file animals-11-00468-s001.pdf]

animals

MDPI

Supplementary Material

The Impact of Whole Sesame Seeds on the Expression of Key-Genes Involved in the Innate Immunity of Dairy Goats

Christina Mitsiopoulou [1], Kyriaki Sotirakoglou [2], Dimitrios Skliros [3], Emmanouil Flemetakis [3] and Eleni Tsiplakou [1,*]

[1] Department of Animal Science, Laboratory of Nutritional Physiology and Feeding, School of Animal Biosciences, Agricultural University of Athens, Iera Odos 75, 118 55 Athens, Greece; chr_mitsiopoulou28@hotmail.com
[2] Department of Natural Resources Management and Agricultural Engineering, Laboratory of Mathematics and Statistics, Department of Natural Resources Management and Agricultural Engineering, Agricultural University of Athens, 11855 Athens, Greece; sotirakoglou@aua.gr
[3] Department of Biotechnology, Laboratory of Molecular Biology, School of Food, Biotechnology and Development, Agricultural University of Athens, Athens, Greece; dsklhros@gmail.com (D.S.), mflem@aua.gr (E.F.)
* Correspondence: eltsiplakou@aua.gr; Tel: +30-210-529-4435, Fax: +30-210-529-4413

Citation: Mitsiopoulou, C.; Sotirakoglou, K.; Skliros, D.; Flemetakis, E.; Tsiplakou, E. The Impact of Whole Sesame Seeds on the Expression of Key-Genes Involved in the Innate Immunity of Dairy Goats. *Animals* **2021**, *11*, 468. https://doi.org/10.3390/ani11020468

Academic Editor: Roberto Rasero
Received: 12 January 2021
Accepted: 8 February 2021
Published: 10 February 2021

Publisher's Note: MDPI stays neutral with regard to jurisdictional claims in published maps and institutional affiliations.

Copyright: © 2021 by the authors. Submitted for possible open access publication under the terms and conditions of the Creative Commons Attribution (CC BY) license (http://creativecommons.org/licenses/by/4.0/).

Table S1. Transcript abundance of several genes in the neutrophils of goats: NOD-like receptor (*NLRC3*), Toll-like receptors 4 (*TLR4*), Nuclear factor kappa B (*NF-KB*), Myeloid-Differentiation-primary response gene 88 (MYD88), Mitogen-Activated Protein Kinase-1 (*MAPK1*), Interleukin 1 Alpha (*IL1A*), Interleukin 1 Beta (*IL1B*), Tumor necrosis factor Alpha (*TNFA*), Tumor necrosis factor Beta (*TNFB*), Interleukin 2 (*IL2*), Interleukin 6 (*IL6*), Signal Transducer and Activator of Transcription 3 (*STAT3*), TIR (Toll/Interleukin-1 Receptor) domain-containing adaptor protein inducing interferon beta (*TRIF*), Interferon Regulatory Factor 3 (*IRF3*), Interferon gamma (*IFNG*), TNF Receptor-associated Factor 3 (*TRAF3*), Interferon Regulatory Factor 5 (*IRF5*), C-C motif chemokine ligand 5 (*CCL5*), Interleukin 8 (*IL8*), Chemokine (C-X-C motif) ligand 16 (*CXCL16*), Heme Oxygenase-1 (*HO1*), Interleukin 10 (*IL10*), Transcription factor JunD (*JUND*) and Conserved Helix-Loop-Helix-Ubiquitous Kinase (*CHUK*) or IKKA relative to the geometrical mean of the references genes (Glyceraldehyde 3-Phosphate Dehydrogenase (*GAPDH*) and Tyrosine 3-monoxygenase/tryptophan 5-monooxygenase activation protein, zeta polypeptide (*YWHAZ*)).

	Diet				Time (Days)				Effect [1]		
	**CON [2]**	**WSS5 [3]**	**WSS10 [4]**	**SEM [5]**	**30**	**60**	**90**	**SEM [5]**	**Diet**	**Time**	**Diet x Time**
NLRC3	0.074	0.063	0.063	0.010	0.056^{A}	0.088^{B}	0.056^{A}	0.007	NS	***	NS
TLR4	0.355	0.313	0.267	0.046	0.357^{A}	0.226^{B}	0.352^{A}	0.033	NS	***	NS
NF-KB	0.299	0.288	0.241	0.026	0.344^{A}	0.268^{B}	0.215^{B}	0.022	NS	***	NS
MYD88	0.742	0.751	0.554	0.114	1.119^{A}	0.488^{B}	0.439^{B}	0.086	NS	***	NS
MAPK1	0.437^{a}	0.450^{a}	0.350^{b}	0.030	0.488^{A}	0.400^{B}	0.349^{B}	0.029	*	**	NS
IL1A	0.379	0.319	0.370	0.058	0.467^{A}	0.370^{AB}	0.233^{B}	0.061	NS	*	NS
IL1B	0.093	0.158	0.109	0.028	0.038^{A}	0.055^{A}	0.267^{B}	0.020	NS	***	NS
TNFA	0.450	0.421	0.400	0.075	0.744^{A}	0.290^{B}	0.238^{B}	0.058	NS	**	NS
TNFB	0.008	0.006	0.006	0.001	0.006^{A}	0.009^{B}	0.005^{A}	0.001	NS	*	NS
IL2	0.002	0.004	0.004	0.001	0.003^{AB}	0.005^{A}	0.002^{B}	0.001	NS	*	NS
IL6	0.0010^{a}	0.0008^{ab}	0.0006^{b}	0.0001	0.0007^{AB}	0.0010^{A}	0.0006^{B}	0.0001	*	*	NS
STAT3	0.352	0.327	0.278	0.031	0.472^{A}	0.245^{B}	0.240^{B}	0.026	NS	***	NS
TRIF	0.008^{a}	0.007^{ab}	0.005^{b}	0.001	0.005^{A}	0.009^{B}	0.006^{A}	0.001	*	*	NS
IRF3	0.090	0.083	0.071	0.008	0.103^{A}	0.066^{B}	0.075^{B}	0.007	NS	***	NS
IFNG	0.029^{a}	0.011^{b}	0.014^{b}	0.006	0.017	0.017	0.020	0.004	*	NS	NS
TRAF3	0.048^{a}	0.034^{b}	0.028^{b}	0.004	0.035	0.040	0.034	0.004	*	NS	NS
IRF5	0.264	0.266	0.221	0.021	0.172^{A}	0.292^{B}	0.287^{B}	0.023	NS	**	NS
CCL5	0.324	0.268	0.221	0.068	0.286^{A}	0.329^{A}	0.198^{B}	0.045	NS	**	NS
IL8	0.685	0.481	0.450	0.094	0.593	0.423	0.599	0.103	NS	NS	NS
CXCL16	0.038	0.043	0.041	0.005	0.030^{A}	0.033^{A}	0.059^{B}	0.004	NS	***	NS
HO1	0.117	0.121	0.089	0.019	0.050^{A}	0.128^{B}	0.142^{B}	0.014	NS	***	NS
IL10	0.039	0.034	0.030	0.006	0.023^{A}	0.024^{A}	0.056^{B}	0.005	NS	***	NS
JUND	0.162^{a}	0.134^{ab}	0.094^{b}	0.017	0.116	0.165	0.110	0.016	*	NS	NS
CHUK	0.050	0.050	0.056	0.003	0.057	0.046	0.052	0.002	NS	NS	NS

Means with different superscripts with small letters (a, b) between the three dietary treatments (CON, WSS5, WSS10) and with capital letters (A, B) between the three sampling time (30, 60, 90) differ significantly ($p < 0.05$). [1] Effect: The dietary treatment (Diet), the sampling time (Time) and the interaction between them (Diet × Time) effects were analysed by ANOVA using a general linear model (GLM) for repeated measures. Post hoc analysis was performed using Duncan's multiple range test. * $p < 0.05$, ** $p < 0.01$, *** $p < 0.001$, NS: not significant [2] CON: Control. [3] WSS5: Whole sesame seeds at 5%. [4] WSS10: Whole sesame seeds at 10%. [5] SEM: Standard error of the mean

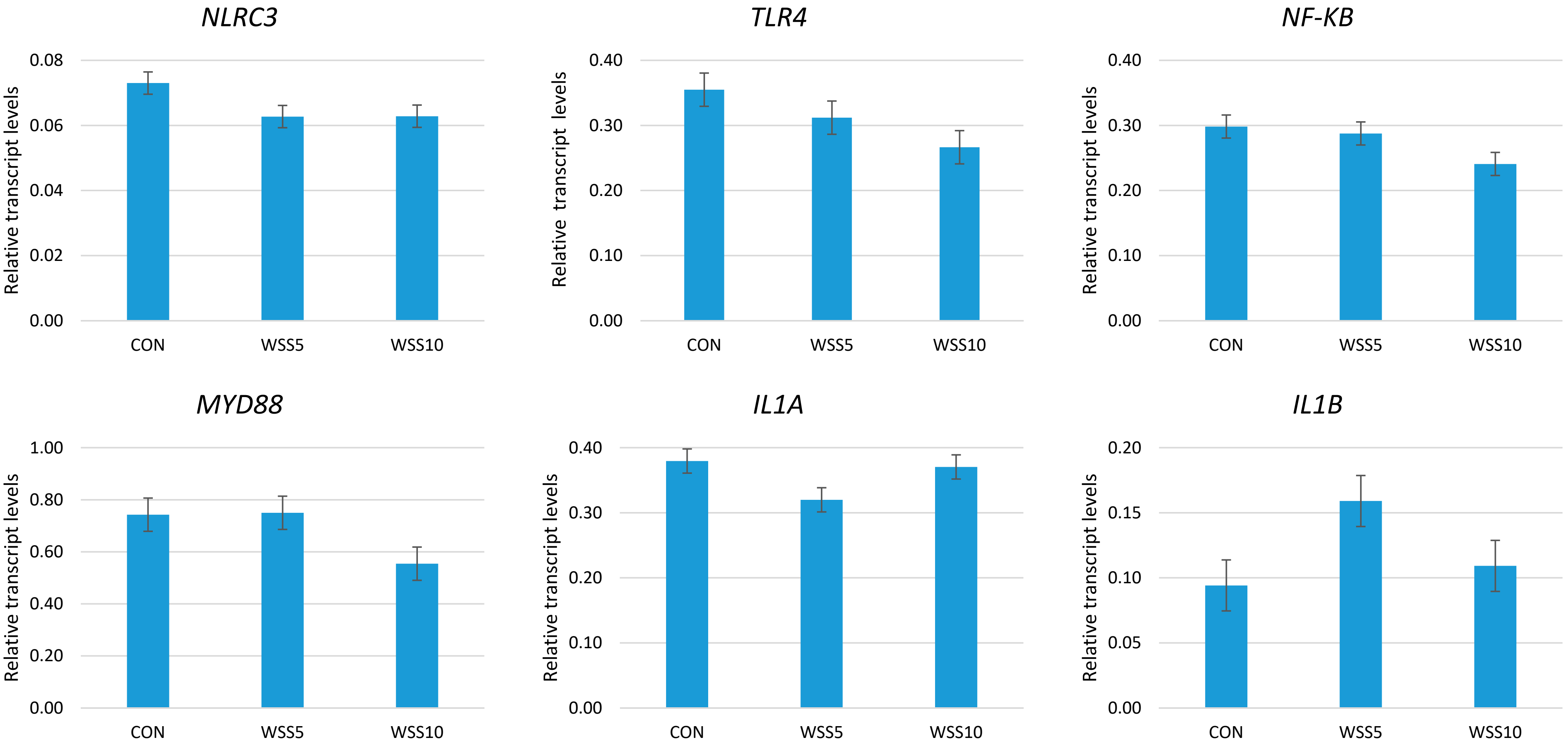
NLRC3
TLR4
NF-KB
MYD88
IL1A
IL1B
Relative transcript levels
0.00
0.02
0.04
0.06
0.08
0.10
0.20
0.30
0.40
0.05
0.15
0.60
0.80
1.00
CON
WSS5
WSS10

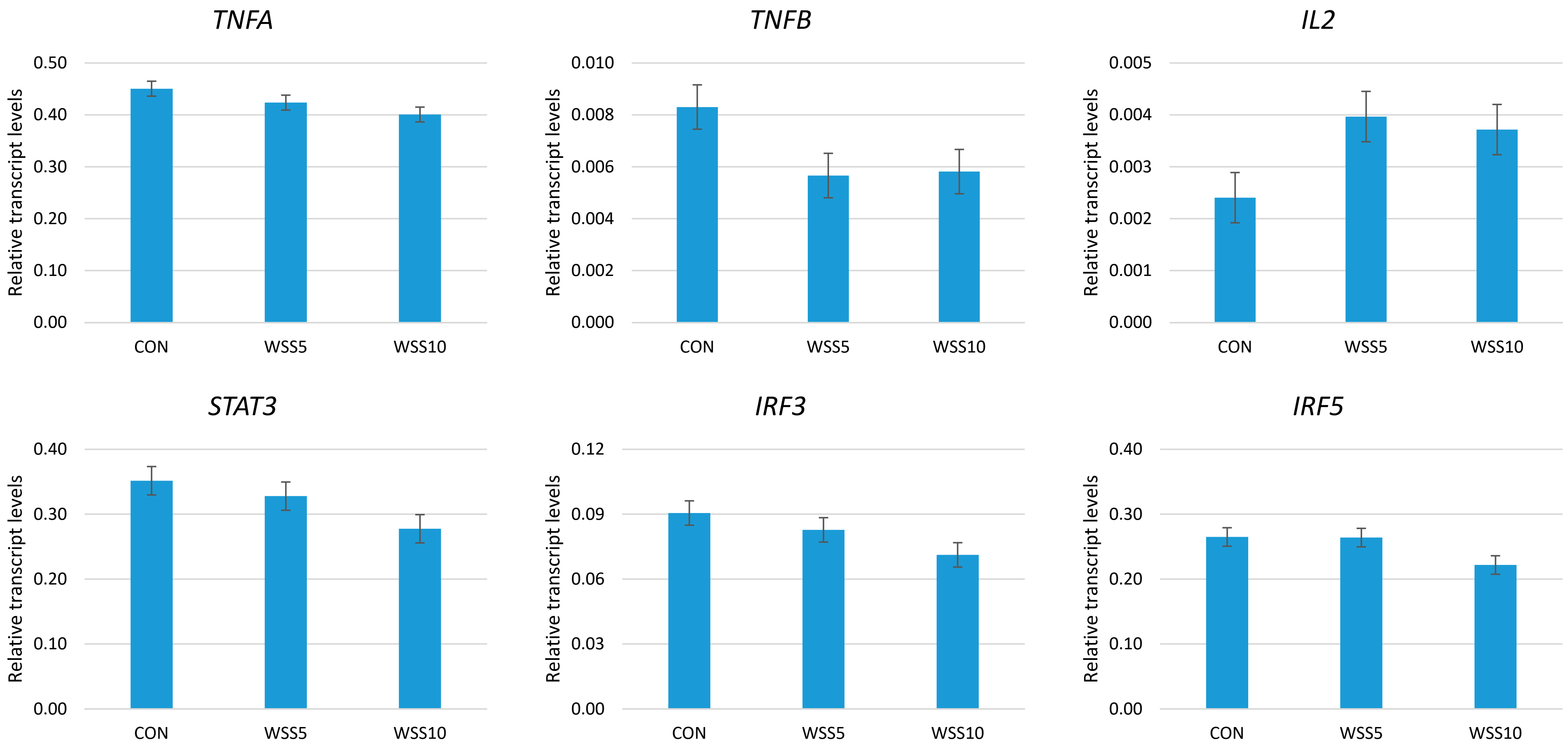
TNFA
TNFB
IL2
STAT3
IRF3
IRF5
Relative transcript levels
0.50
0.40
0.30
0.20
0.10
0.00
0.010
0.008
0.006
0.004
0.002
0.000
0.005
0.004
0.003
0.002
0.001
0.12
0.09
0.06
0.03
CON
WSS5
WSS10

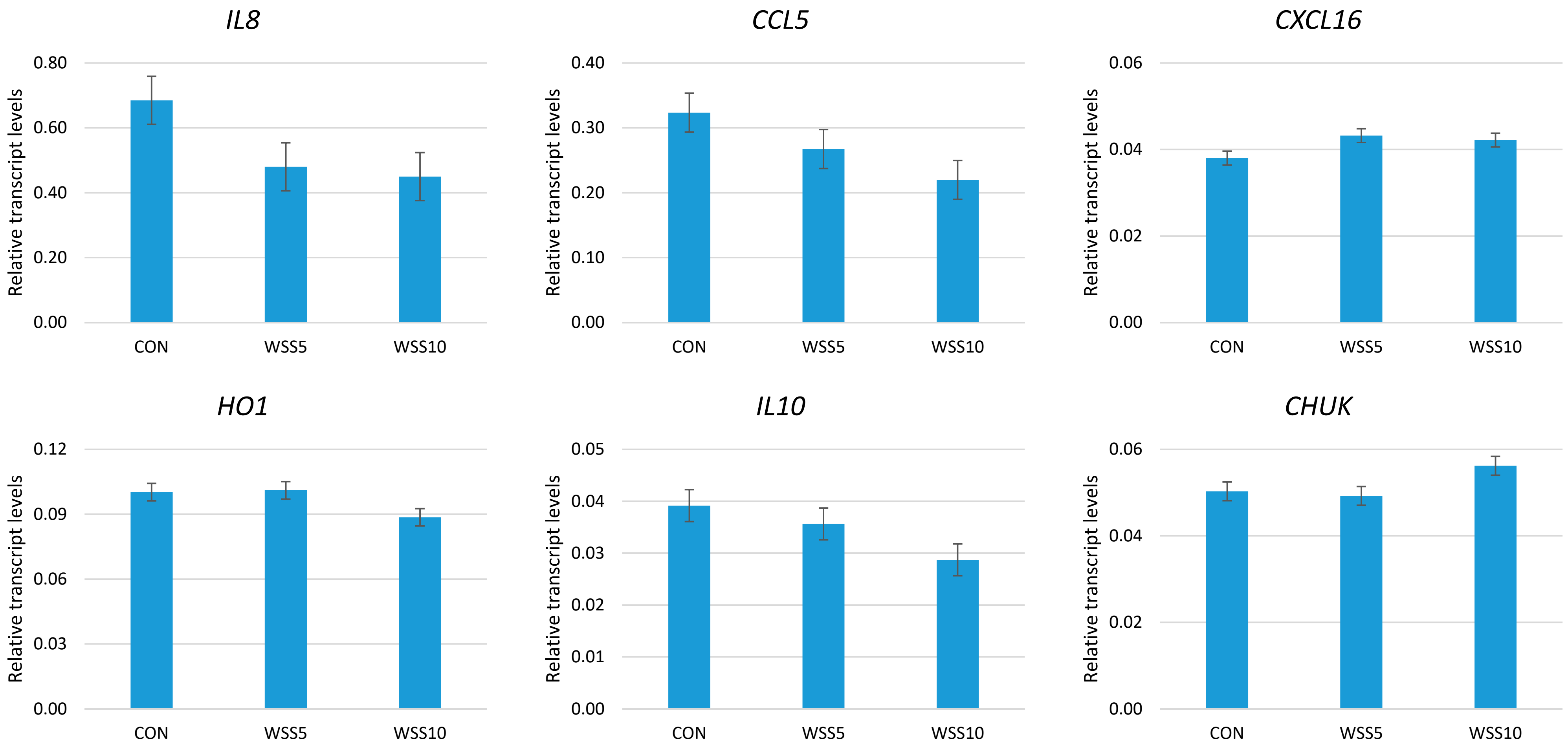

Figure S1. The Transcript abundance of several genes in the neutrophils of goats. Bars represent means ± SEM of each (n = 8) of the three dietary treatments; CON: control, basal diet; WSS5: basal diet + 5% whole sesame seed; WSS10: basal diet + 10% whole sesame seed in goats. The analysis of variance (ANOVA) using a general linear model (GLM) for repeated measures revealed that for these genes there was not significant difference between the three dietary treatments ($p > 0.05$).
